# What differentiates youths who use e-cigarettes from those who smoke traditional tobacco products?

**DOI:** 10.1186/s12889-022-13673-0

**Published:** 2022-07-15

**Authors:** Hugo Torregrossa, Bertrand Dautzenberg, Pierre Birkui, Nicole Rieu, Marie-Dominique Dautzenberg, Maria Melchior, Murielle Mary-Krause

**Affiliations:** 1 Sorbonne Université, INSERM, Institut Pierre Louis d’Épidémiologie Et de Santé Publique (IPLESP), ERES, 75012 Paris, France; 2 Service de Pharmacologie, Hôpital Pitié-Salpêtrière, AP-HP, 75013 Paris, France; 3 Consultation de Tabacologie, Institut Arthur Vernes, 75006 Paris, France; 4 Paris Sans Tabac (PST), 75007 Paris, France; 5 Rectorat de L’Académie de Paris, 75019 Paris, France

**Keywords:** Electronic cigarettes, Tobacco, Smoking, Adolescents, Youth, Profiles

## Abstract

**Background:**

Electronic cigarette (e-cigarette) use has spread among adolescents in many countries, however users’ characteristics are not well known. We aimed to compare characteristics of exclusive e-cigarette users to those of exclusive tobacco users and dual users.

**Methods:**

Data come from a representative sample of 11–19 years old students in Paris, surveyed each year between 2013 and 2017. Current e-cigarette and tobacco use were ascertained in the preceding 30 days. Data were analyzed using random intercept multinomial logistic regression models, exclusive tobacco smokers being the reference group.

**Results:**

Among the 17,435 students included, 2.3% reported exclusive e-cigarette use, 7.9% exclusive tobacco use and 3.2% dual e-cigarette and tobacco use. Compared to exclusive tobacco smokers, e-cigarette users were: a) less likely to use cannabis (adjusted Odds-Ratio (aOR) = 0.15, 95% confidence interval (95% CI) = 0.09–0.25); b) more likely to initiate smoking with an e-cigarette or a hookah rather than traditional cigarettes (aOR = 2.91, 95% CI = 1.74–4.87 and aOR = 15.99, 95% CI = 8.62–29.67, respectively). Additionally, exclusive e-cigarette users are younger with an aOR = 0.29 (95% CI = 0.17–0.49) among 13–15 years and aOR = 0.11 (95% CI = 0.06–0.21) among > 17 years as compared to 11–13 years. The probability of being an exclusive e-cigarette user is lower among participants whose best friend smokes tobacco (aOR = 0.30, 95% CI = 0.20–0.44). Exclusive tobacco users and dual users have similar profiles.

**Conclusions:**

Adolescents who only used e-cigarettes had intermediate levels of risk compared to nonusers and those who used tobacco and/or e-cigarettes, suggesting that e-cigarettes use extends to young people at low-risk of using tobacco products.

**Supplementary Information:**

The online version contains supplementary material available at 10.1186/s12889-022-13673-0.

## Background

In many countries, adolescent use of electronic cigarettes (e-cigarettes) is an emerging public health issue [1, 2]. The past 30 days prevalence among US students increased from 11.0% to 25.4% between 2017 and 2019 in 12^th^ grade, from 8.2% to 20.2% in 10^th^ grade and from 3.5% to 9.0% in 8^th^ grade [3]. Similar increases have occurred in European and Asian countries [4, 5] with 14% of European 14–15 year olds who have used e-cigarettes in the last 30 days in 2019 [6]. In France, e-cigarettes were released in 2010 [7] and since March 2014 the law forbids their sale to youths who are underage (< 18 years). According to a national French survey [8], 1.2% of 15–24 year olds reported being daily users in 2016 vs 2.1% in 2014 and e-cigarette experimentation seems to remain stable at a high percentage. At 17 years old, 52.4% have experimented e-cigarette, 16.8% declared a use in past 30 days and 1.9% notified a daily use [9].

Examining factors associated with e-cigarette use among secondary school students is important for several reasons. First, secondary school students are at the forefront of substance use trends that ultimately become prevalent in the population [10], suggesting that the characteristics of adolescent e-cigarette users of today could be indicative of who may be most likely to use these products in the future. A recent study showed that e-cigarette susceptibility measures appear to predict e-cigarette use among youth 1 year later with odds-ratio close 3 [11]. Second, even though e-cigarettes have become more widely available and accessible nationwide [12], particularly in retail outlets near college campuses [13], predictive factors of the onset of e-cigarette use are not well known. A growing literature has identified varying factors associated with smoking intention, such as parental or peer smoking, exposure to secondhand smoke inside or outside the home, pro-tobacco advertising, and school connectedness [13–15], binge drinking, cannabis use and other illicit drug use, low educational attainment, and truancy [14–17]. However, relationships between e-cigarette use and these potential risk factors are not well known even if an increased risk of individual substance use behaviors was demonstrated among e-cigarette users, such as cigarette smoking and cannabis use, especially among older adolescents and young adults [18–20]. Numerous studies had studied the impact of e-cigarette on cigarette smoking in the future among young people. Some studies stated that students using e-cigarettes were more likely to start smoking tobacco than others in US [21] but also in Europe [22, 23], but exclusion of smokers could be a bias in studies, among others. A meta-analysis showed that majority of studies reporting a positive association between vaping and subsequent smoking initiation have critical or serious risk of bias [24]. A study considering all students showed that e-cigarettes in adolescents are not a significant “gateway” to tobacco but in fact diverting adolescents from cigarettes, nevertheless maintaining the total nicotine use prevalence [25].

The situation is different in France where less than 1% of 18–75 year olds people who tried vaping never smoked tobacco [26]. It is rare that a regular e-cigarette user has never smoked traditional cigarettes before. Some studies identified factors associated with e-cigarette use in young adulthood such as male gender and cigarette smoking [27]. Nevertheless, these studies are established on young adults (22–25 years), and few studies differentiated dual users from exclusive e-cigarette users, and examined a wider range of risk factors. Thus, there is need to obtain greater clarity regarding adolescents’ e-cigarette use in relation to a wider range of factors associated with smoking intention such as sex, age, friends’ and siblings’ smoking status, smoking perception, parental ban on smoking, the first initiated tobacco-related product, cannabis use, binge drinking and parental smoking [18–20, 28]. A recent study among the National Youth Tobacco Survey (NYTS) in US middle school and high school describes the prevalence of youth tobacco product use and associated factors [29], but no comparison was performed among different user profiles.

The current study aims to determine: (1) whether there is a gradient in terms of substance-related risk behaviors between the four following profiles: nonusers, exclusive e-cigarettes users, exclusive tobacco users and dual users; (2) whether the first initiated tobacco-related product predicts later patterns of use.

## Methods

### Sample

Data was collected between 2013 and 2017 through the “Paris sans Tabac” (PST, Paris without tobacco) study, a yearly repeated cross-sectional survey conducted among secondary school students in Paris, France. Parisian middle school (*n* = 9,017) and high school students (*n* = 8,418) were selected using random sampling with quotas applied to balance school types (private vs. public) and class levels in middle school and high school. Each year, after selection of the school and the classroom, all students of the selected classes were invited to answer to the questionnaire. The study sample was constructed to correspond to approximately 2% of all Parisian middle and high school students, with an additional 10% to take into account school absenteeism, i.e. a total of 2.2% of all students. So, each year, approximately 3,500 students were included in the study. Because of the low percentage of Parisian students included in our study (2.2%), the probability of the same student attending two consecutive years is almost zero.

According to different surveys in France, daily smoking prevalence has been estimated to be 0.6% at 11 years old, 4% at 13 years old, 19% at 15 years old in 2010 [30], 31% at 16 years old in 2011 [31] and 32% at 17 years old in 2011 [32]. In a sample of 3,500 students per year, we expect more than 1000 smokers and a 3% difference in the prevalence of smoking can be highlighted with a type I error of 0.05 and a power of 80%.

Supplementary Table 1 shows the distribution of the sample for each year, type of school and number of smokers for each type of smoker, showing an overrepresentation of the high school students in 2014 compared with other studied years (< 0.0001).

### Measures

During class hours, students self-completed questionnaires (questionnaire translated in English in Supplementary Fig. 1) to report their demographic, individual, family smoking characteristics as well as their tobacco, alcohol, cannabis and e-cigarette use. Past 30 days e-cigarette and tobacco use were assessed with the following item: “During the past 30 days, did you use: a cigarette/ hookah/ e-cigarette/ rolled cigarettes/ menthol cigarettes/ perfumed cigarettes/ pipes / cigars, cigarillos?” (multiple replies are possible). Hookah, also called shisha, water pipe or narghile, is one of the few forms of tobacco use which consists of the inhalation of tobacco smoke after it passes through water through a bowl with a hose that the smoker uses to breath in the smoke. Tobacco use groups were categorizedas nonusers (those who have not used an e-cigarette or any form of tobacco in the past 30 days), exclusive e-cigarette use (those who have used only e-cigarettes, with or without nicotine, in the past 30 days), exclusive tobacco smokers (those who have used any form of tobacco including cigarettes, pipes, cigars, cigarillos and hookah, excluding e-cigarettes, in the past 30 days) and dual users (those who have used both e-cigarettes and any form of tobacco in the past 30 days).

Studied characteristics as potentially associated with electronic cigarette and tobacco use included the first initiated tobacco-related product, age, sex, grade, cannabis use /alcohol use, hookah ever experienced, best friend’s/brother’s or sister’s tobacco use status, perception of peer smoking, parental ban on smoking, and survey year. Data on current use of cannabis was collected by asking “Have you ever smoked anything else than tobacco?” and “During the past 30 days, did you use cannabis?”. Alcohol consumption was assessed via the questions “Do you drink alcohol sometimes?” and “If yes, do you drink at least once per month?”. The perception of peer smoking derived from the number of estimated smokers out of 10 students. This information was used to take into account the propensity to stigmatize smokers strongly decreased with increased use of tobacco, especially among former regular smokers [33]. As tobacco initiation with hookah was found more strongly associated with current smoking than other tobacco products in our study, we have distinguished this product from the others.

### Analytic approach

Characteristics of subjects in each group tobacco and/or e-cigarette use were compared using chi-square tests.

Given missing data on several study covariates (maximum 12% for the variable characterizing the best friend’s smoking), missing data were imputed using a fully conditional specification (FCS) with 20 iterations [34].

Associations between each group of tobacco-related products use and covariates was estimated using a mixed-effects multinomial model with a random effect, accounting for sampling and imputation uncertainty. The choice of a mixed effect regression model was justified by the clustered sampling design [35, 36]. Indeed, we wanted to take into account a random-effect, the classroom defining the cluster, because we assumed that there is a variability between classrooms.

In order to take into account the first initiated tobacco-related product notified only among smokers, we conducted two separate analyses: the first one compared the three tobacco-related product user groups, and the second one compared nonusers to exclusive e-cigarette users. For the sake of robustness and parsimony with respect to statistically selected covariates, variable selection procedures were conducted using random forests and lasso regularization [37]. These complementary methods yielded similar results to the main analyses and led us to retain a set of covariates consisting of age, the use of cannabis, the first initiated tobacco-related product and the best friend’s smoking status. Exchanges with a tobacco control expert enabled us to add four other characteristics to our initial selection: survey year, alcohol consumption, perception of peer smoking and parental ban on smoking. In order to determine the degree of proximity or distance of consumption profiles between each other, we calculated the AUC (Area under Roc curve) on each logistic binary submodel (exclusive tobacco smokers vs. exclusive e-cigarette users and exclusive tobacco smokers vs. dual users) with a tenfold cross validation to control overfitting.

An exploratory sensitivity analysis was performed to examine the robustness of our findings using only available data (without imputing missing data). Moreover, a second sensibility analysis comparing exclusive e-cigarette users to non-users was performed after removal of the former smokers (*n* = 342).

Except for random forests and lasso regularization methods computed with R, all analyses were performed with SAS 9.4. The ethics committee of Sorbonne University (Paris) did not find any ethical problems (reference number CER-2022–011). As study questionnaires were completely anonymous, with no possibility to identify participants in the study among the 190,100 Parisian students aged 11–19 years, this study is outside the framework of the data protection regulation. According to French regulations, signed informed consent was not necessary for our study, but a non-opposition agreement was proposed. Under the supervision of the Paris School authority, parents and/or students age 18 years and older were informed and could refuse participation; parents could refuse on behalf of minor students’, and students age 18 years and older could refuse for themselves.

## Results

Among the 17,435 students included in the study between 2013 and 2017, with a median age of 16 years, 2,317 (13.3%) reported smoking tobacco and/or e-cigarette use in the past 30 days: 2.3% (*n* = 392) students reported exclusive e-cigarette use, 7.9% (*n* = 1,370) exclusive tobacco use and 3.2% (*n* = 555) dual use (Table 1). Among these subjects, only 8.0% started vaping with the e-cigarette, 10.0% started smoking with a hookah and 82.0% with tobacco. Among the exclusive e-cigarette users, the first product used was e-cigarette for 38.5%, hookah for 16.0% and cigarette or another smoked tobacco apart from hookah for 45.5%. Among the exclusive tobacco smokers, the first product used was e-cigarette for 2.0%, hookah for 8.2% and cigarette or another smoked tobacco apart from hookah for 89.8%. Among dual users, the first product used was e-cigarette for 3.6%, hookah for 10.7% and cigarette or another smoked tobacco apart from hookah for 85.7%.

**Table 1:** Characteristics of students by tobacco-related use profiles (PST study, *n* = 17,435, 2013–2017)

	Non users	Exclusive e-cigarette users	Exclusive tobacco users	Dual users	*P* value^a^
	(*n* = 15,118)	(*n* = 392)	(*n* = 1370)	(*n* = 555)	
	*n (%)*	*n (%)*	*n (%)*	*n (%)*	
First initiated tobacco-related product					
Tobacco (except hookah)	NA	151 (38.5)	1189 (86.8)	455 (82.0)	< 0.0001
E-cigarette	NA	128 (32.7)	27 (2.0)	19 (3.4)	
Hookah	1137 (7.5)	53 (13.5)	108 (7.9)	57 (10.3)	
Age					
≤ 13 years	6157 (40.7)	146 (37.2)	63 (4.6)	34 (6.1)	< 0.0001
14–17 years	5554 (36.7)	187 (47.7)	587 (42.9)	295 (53.2)	
> 17 years	3344 (22.1)	56 (14.8)	715 (52.2)	226 (40.7)	
Sex					
Female	7621 (51.5)	173 (45.5)	767 (57.0)	277 (50.6)	< 0.0001
Male	7174 (48.5)	207 (54.5)	578 (43.0)	270 (49.4)	
Grade					
Middle school					< 0.0001
1^st^ level	2183 (14.4)	20 (5.1)	9 (0.7)	6 (1.1)	
2^nd^ level	2085 (13.8)	40 (10.2)	15 (1.1)	8 (1.4)	
3^rd^ level	2243 (14.8)	98 (25.0)	51 (3.7)	27 (4.9)	
4^th^ level	1998 (13.2)	77 (19.6)	100 (7.3)	57 (10.3)	
High school					
1^st^ level	2164 (14.3)	67 (17.1)	325 (23.7)	149 (26.8)	
2^nd^ level	2196 (14.6)	48 (12.2)	427 (31.2)	159 (28.6)	
3^rd^ level	1517 (10.0)	38 (9.7)	335 (24.4)	122 (22.0)	
Preparation for engineering competitions	391 (2.6)	1 (0.3)	71 (5.2)	17 (3.1)	
Others	341 (2.3)	3 (0.8)	37 (2.7)	10 (1.8)	
Cannabis use					
Never experienced	10,489 (69.4)	271 (69.1)	278 (20.3)	77 (13.9)	< 0.0001
Already experienced	1305 (8.6)	63 (16.1)	490 (35.8)	186 (33.5)	
At least one time per month	685 (4.5)	32 (8.2)	560 (40.9)	274 (49.4)	
Alcohol consumption					
Never drink	8907 (58.9)	159 (40.6)	167 (12.2)	47 (8.5)	< 0.0001
Drink sometimes but less than once a month	2625 (17.4)	96 (24.5)	261 (19.1)	99 (17.8)	
Drink more than once a month	2957 (19.6)	110 (28.1)	860 (62.8)	380 (68.5)	
Hookah ever experienced					
No	10,455 (69.2)	159 (40.6)	157 (11.5)	51 (9.2)	< 0.0001
Yes	4101 (27.1)	207 (52.8)	1185 (86.5)	500 (90.1)	
Smoking status of the best friend					
Non-smoking	10,266 (67.9)	225 (57.4)	250 (18.3)	83 (15.0)	< 0.0001
Smoker/Former smoker	2792 (18.5)	111 (28.3)	957 (69.9)	416 (75.0)	
Smoking status of brothers/sisters					
Non-smoking	11,770 (80.6)	272 (72.2)	734 (54.7)	306 (56.2)	< 0.0001
Smoker/Former smoker	2829 (19.4)	105 (27.8)	608 (45.3)	239 (43.8)	
Perception of peer smoking rate^b^					
None	4516 (29.9)	60 (15.3)	24 (1.8)	10 (1.8)	< 0.0001
Between 1 and 5	7334 (48.5)	244 (62.2)	734 (53.6)	261 (47.0)	
Between 6 and 10	2491 (16.5)	70 (17.9)	572 (41.8)	274 (49.4)	
Parental ban on smoking					
No	6308 (41.7)	142 (36.2)	734 (53.6)	286 (51.5)	< 0.0001
Yes	8216 (54.5)	240 (61.2)	594 (43.4)	258 (46.5)	
Survey year					
2013	2790 (18.5)	43 (11.0)	347 (25.3)	99 (17.8)	< 0.0001
2014	2888 (19.1)	109 (77.8)	224 (16.4)	142 (25.6)	
2015	3028 (20.0)	96 (24.5)	273 (19.9)	120 (21.6)	
2016	3081 (20.4)	79 (20.2)	284 (20.7)	103 (18.6)	
2017	3331 (22.0)	65 (16.6)	242 (17.7)	91 (16.4)	

^a^ *p* values were computed using Pearson Khi 2 tests

^b^ Out of 10 students, how many smoke every day ?

### Exclusive e-cigarette users vs. exclusive tobacco users (Table 2)

**Table 2:** Factors associated with tobacco-related use profiles (PST study, *n* = 2,317, 2013–2017): Multivariable multinomial analysis

	Exclusive e-cigarette users vs exclusive tobacco users *OR (95% CI)* ^a^	dual users vs exclusive tobacco users *OR (95% CI)* ^a^
First initiated tobacco-related product		
Tobacco (except hookah)	1	1
E-cigarette	2.91 (1.74–4.87)***	1.41 (0.96–2.06)
Hookah	15.99 (8.62–26.67)***	1.76 (0.91–3.41)
Age		
≤ 13 years	1	1
14–17 years	0.29 (0.17–0.49)***	0.77 (0.45–1.29)
> 17 years	0.11 (0.06–0.21)***	0.42 (0.25–0.72)***
Cannabis use		
Never experienced	1	1
Already experienced	0.34 (0.22–0.52)***	1.51 (1.07–2.13)*
At least one time per month	0.15 (0.09–0.25)***	1.90 (1.35–2.67)***
Alcohol consumption		
Never drink	1	1
Drink sometimes but less than once a month	0.73 (0.44–1.20)	1.18 (0.76–1.85)
Drink more than once a month	0.63 (0.40–0.99)*	1.51 (1.03–2.23)*
Smoking status of the best friend		
Non-smoking	1	1
Smoker/Former smoker	0.30 (0.20–0.44)***	1.31 (0.96–1.78)
Perception of peer smoking rate^b^		
None	1	1
Between 1 and 5	0.35 (0.16–0.76)***	0.87 (0.38–2.00)
Between 6 and 10	0.26 (0.12–0.60)**	1.14 (0.49–2.63)
Parental ban on smoking		
No	1	1
Yes	1.48 (1.00–2.18)	0.93 (0.72–1.20)
Survey year		
2013	1	1
2014	4.23 (2.11–8.49)***	2.56 (1.64–4.00)***
2015	2.37 (1.18–4.74)*	1.78 (1.14–2.75)*
2016	2.46 (1.22–4.97)*	1.50 (0.95–2.35)
2017	2.41 (1.12–5.19)*	1.44 (0.90–2.29)

^a^ Adjusted Odds ratio (OR), 95% confidence interval (95% CI), * *p* < 0.05 ** *p* < 0.01 ***p < 0.001

^b^ Out of 10 students how many smoke every day ?

Multivariable multinomial logistic regression analyses showed that, compared with those who began smoking with a tobacco product, those who began with an e-cigarette have a higher probability of becoming exclusive e-cigarette users than exclusive tobacco users (adjusted odds-ratio (aOR) = 2.91; 95% confidence interval (95% CI) = 1.74–4.87). In the same way but more importantly, compared with those who began smoking with a tobacco product, students who began smoking with a hookah are more likely to become exclusive e-cigarette users than exclusive tobacco users (aOR = 15.99; 95% CI = 8.62–26.67). A gradual “protective” association is found between age and cannabis use and being an exclusive e-cigarette, rather than tobacco user. Other covariates associated with students’ smoking profile included survey year, alcohol consumption, best friend’s tobacco smoking status, perception of peer smoking and parental ban on smoking.

### Dual users vs exclusive tobacco users (Table 2)

Contrary to the comparison between exclusive e-cigarette users and exclusive tobacco users, no significant association was found comparing dual users and tobacco users about first initiated tobacco-related product.

Only students’ age and cannabis use significantly distinguished exclusive tobacco smokers from dual users: compared with students younger than 14 years, students 17 years or older had a smaller likelihood of being dual users (aOR = 0.42; 95% CI = 0.25–0.72). Students who experimented cannabis or used cannabis in the preceding month were more likely to be dual users than exclusive tobacco users (aOR = 1.51; 95% CI = 1.07–2.13 and aOR = 1.90; 95% CI = 1.35–2.67, respectively).

Additional analyses focusing on both exclusive and dual tobacco users showed that 73% were daily smokers and that dual users consumed slightly more cigarettes than exclusive tobacco users (median 7 vs. 6, *p* < 0.001, respectively). There were no statistically significant differences between these two groups in terms of age of onset of smoking/cannabis use or smoking dependence.

### E-cigarette users vs nonusers (Table 3)

**Table 3:** Factors associated with e-cigarette use compared to nonuse of e-cigarette and tobacco products (PST study, n = 15,510, 2013–2017): Multivariable multinomial analysis

	Exclusive e-cigarette users vs Nonusers *OR (95% CI)* ^a^
Age	
≤ 13 years	1
14–17 years	0.65 (0.47–0.90)**
> 17 years	0.25 (0.16–0.38)***
Cannabis use	
Never experienced	1
Use in the past month	0.87 (0.57–1.32)
Alcohol consumption	
Never drink	1
Drink sometimes but less than once a month	1.71 (1.29–2.27)***
Drink more than once a month	1.56 (1.16–2.10)***
Smoking status of the best friend	
Non-smoking	1
Smoker/Former smoker	1.39 (1.05–1.84)*
Perception of the smoking rate^b^	
None	1
Between 1 and 5	2.10 (1.49–2.97)***
Between 6 and 10	1.78 (1.14–2.77)*
Hookah ever experienced	
No	1
Yes	3.36 (2.60–4.35)***
Survey year	
2013	1
2014	2.64 (1.62–4.29)***
2015	2.48 (1.52–4.02)***
2016	1.98 (1.21–3.28)**
2017	1.64 (0.99–2.74)

^a^ Adjusted Odds ratio (OR), 95% confidence interval (95% CI), * *p* < 0.05 ** *p* < 0.01 ***p < 0.001

^b^ Out of 10 students how many smoke every day ?

In additional analyses, we compared exclusive e-cigarette users with nonusers, previously found to have a lower risk profile than tobacco users. Our imputed sample included 15,510 students across the five study waves: 97.5% (n = 15,118) students reported no cigarette use and 2.5% (n = 392) exclusive e-cigarette use.

Multivariable multinomial logistic regression analyses showed that, compared with those who did not experience the hookah, those who did were more likely to become exclusive e-cigarette users rather than nonusers (aOR = 3.36; 95% CI = 2.60–4.35). Students who were older than 17 years had a smaller likelihood of becoming exclusive e-cigarette users than students who were 13 years or less (aOR = 0.25; 95% CI = 0.16–0.38). Similarly, the best friend’s smoking status, survey year, alcohol use and perception of peer smoking were also significantly associated with exclusive e-cigarette use rather than no use.

All exploratory sensitivity analyses showed consistent results in the level of significance, aORs estimates and slightly larger magnitude of aORs.

## Discussion

This study aimed to examine possible differences in substance-related risk behaviors between nonusers, exclusive e-cigarettes users, exclusive tobacco users and dual users, and whether the first initiated tobacco-related product predicts later patterns of use. Studying a representative sample of adolescents living in Paris, we found that youths who only used e-cigarettes had intermediate levels of risk between nonusers and those who used tobacco and/or e-cigarettes. This suggests that e-cigarettes extend among young people at low-risk of using tobacco products. Dual users and tobacco-only users accumulated risk factors as demographic factors (age, best friend smoker, parental ban, perception of peer smoking rate) and risk behaviors (cannabis, alcohol) compared exclusive e-cigarette users and nonusers. Among the three profiles smoking and/or vaping, our study identifies two very distinct profiles: students who only use e-cigarettes are different from tobacco users and dual users. Differences are much smaller between tobacco user and dual user than between e-cigarette users and tobacco users. Nevertheless, dual users are older and likely to use cannabis and alcohol than tobacco users. These results are interesting insofar as the factors associated with e-cigarettes use are for the most part also associated with tobacco use [38, 39]. In addition, long-term health effects are probably not the same according the use of e-cigarette with or without tobacco smoking [40, 41] with one recent study suggesting more negative health effects for dual use than cigarette smoking alone [42].

### Strengths and limitations

The principal strength of this study is its size and representative nature. Indeed, more than 17,000 adolescents drawn randomly were studied over 5 years. Schools are spread throughout Paris and is socioeconomically diverse.

The inclusion of several questions about the experimentation and frequency of use of psychoactive substances (cannabis, alcohol), as well as the first initiated tobacco-related product and peers’ smoking status allowed us to take into account important confounding factors. Unfortunately, the measure of e-cigarette use we included does not specify the types of products that are available [1, 2]. In addition, sensitivity analyses showed that our inferences were robust to biases possibly introduced by missing data.

Our study has several limitations. First, the design is cross-sectional, which precludes any conclusions about the causal relationships between e-cigarette use and identified risk behaviors, even though the initiation of tobacco-related products temporally preceded past 30-day cigarette smoking and e-cigarette use. Nevertheless, we can highlight factors associated with the use of tobacco-related products. In addition, as only Parisian youths are included in this study, results cannot be generalized to youths of same age in other geographical areas of France, or from other countries or regions than the present sample. In fact, Parisians have the lowest smoking rate in France (23.6% of daily smokers vs. 31.3% in Metropolitan France in 2014) [43]. Second, there are subgroups of French youths who are not included in our sample, such as students who are home schooled or have dropped out of school (1.8% of out-of-school Parisian students aged 11–17 in 2015 [44]) or were absent on the day when data were collected (about 90% of response rate). Home-schooled youths are less likely to engage in substance use behaviors [45], while those who drop out or are often absent from school are more likely to engage in substance use and other risk behaviors [46].

Third, all measures are based on self-reports, and while prior work has found that such measures are reliable and valid, misclassification and under-reporting of sensitive behaviors such as substance use can occur [18, 28, 47, 48]. In this study, no adjustment was made to correct for under-reporting; thus, results may be conservative and under-report the actual prevalence of tobacco and electronic cigarette use even though measurement error is probably compensated for by the large sample. In the present research, we controlled for a number of variables correlated with e-cigarette/tobacco use such as index disposition to smoke cigarettes (eg. age, peers’ smoking status, parental support), and we found that the role of the first initiated tobacco-related product was significant. Some potential confounding factors were not included in the questionnaire such as parental education, illicit drug use, students’ psychological characteristics (depression, anxiety, impulsivity) [49]. Fourth, we had an overrepresentation of high-school students in 2014, compared with other studied years, a period where there are more smokers than in middle schools. Nevertheless, there is no reason that smoker characteristics differed over the limited studied period. Finally, we had no information regarding the type of e-cigarette used (level of nicotine, adjustable power, type of flavors…) or the context of use, which would likely modify the factors associated with e-cigarette users. Nevertheless, comparisons between groups of tobacco-related product users give some confidence results as comparisons are made in the same population.

### Comparison with prior literature

The use of an e-cigarette as the first initiated tobacco-related product was associated with exclusive e-cigarette use. Hookah use is to a lesser extent also significantly associated with current use of e-cigarettes without tobacco. Moreover, lower-risk adolescents (in terms of alcohol, cannabis consumption, age and peers’ smoking status) seem to be more attracted by e-cigarette. This phenomenon is confirmed by studies regarding reasons for e-cigarette use that indicate that curiosity, fashionability, and social popularity are salient motives for use [50–52]; these are likely to be prevalent among lower-risk adolescents. The availability of attractive flavorings and the perception that e-cigarettes are healthier than cigarettes [2, 18, 52] would also tend to encourage experimentation among youths who are less influenced by a desire to take risks, be rebellious or unconventional, which characterize adolescent cigarette smokers [20, 53]. Moreover, we observed that the prevalence of e-cigarette use fell over time relative to non-users or users of tobacco [54].

As observed in other studies, but with a much larger number of participants and a more representative sample, we found that exclusive e-cigarette users have an intermediate tobacco risk compared to non users and tobacco smokers with or without e-cigarette use [53, 55–57], and exclusive and dual tobacco users have similar profiles [1, 56, 57]. Moreover, e-cigarette users are divided into two groups in our study, those who do or do not use tobacco, with the group of dual users more numerous (555 (24%) vs. 392 (17%)) which is also the case in other studies [1, 56–58]. Friends’ and siblings’ smoking status are correlated with dual use among adolescents and young adults [55–58]. Our study investigated some factors which could be associated with tobacco use such as parental ban [59, 60] and perception of peer smoking rate [61, 62], neither of which have been previously studied to differentiate consumption profiles. Moreover, tobacco initiation with hookah has never been studied to investigate difference in tobacco use profile, even though it has been shown that hookah is popular among young people [63, 64].

The fact that exclusive e-cigarette users have intermediate levels of risk between nonusers and dual users raises the possibility that e-cigarettes are used by adolescents who otherwise would not use tobacco products. It was shown that curiosity was the most commonly reported reason among current exclusive e-cigarette users [65], adolescents using these products primarily for recreational purposes rather than a means to help reduce cigarette smoking [10] with majority of young exclusive e-cigarette users using e-liquids without nicotine and percentage of nicotine users being higher among dual users [10, 66]. Moreover, e-cigarette can also appear as a more interesting product for potential smokers. Results from the National Youth Tobacco Survey (NYTS) show that adolescents reported the highest harm perception with cigarettes and the lowest with e-cigarettes [67], the latter being a way to use nicotine without being exposed to the toxic substances contained in cigarettes [7]. In addition, our study began at the very beginning of the arrival of the e-cigarette among young French people in 2013–2014, with consumption that began among the youngest as an experimentation product [65]. Note that during the study period, 17-year-olds who were regular smokers has gone from 32% in 2014 to 25% in 2017 [68] and tobacco experimentation among middle school students decreased from 27.8% in 2014 to 21.2% in 2018 [69], which is not compatible with the concept of e-cigarette as a gateway to tobacco and rather evoked an effect of substitution.

## Conclusion

This study showed that e-cigarette use extends even to young people at low-risk of using tobacco products and that tobacco users with or without e-cigarette accumulated risk factors. As uncertainties regarding e-cigarette are not yet settled, this study increases our understanding of e-cigarette use to improve youth tobacco control policy. Indeed, it is important to target young people in prevention program, not only to address cigarette smoking, but also to address e-cigarette use use. Because recent studies showed possible toxic effects of electronic cigarettes and vaping in adolescents [70], public health efforts must urgently be implemented to delay or eliminate e-cigarette initiation, help current users to stop, and to stop the spread of nicotine vaping among adolescents.

## Supplementary Information

**Additional file 1:** **Supplementary Figure 1.** Students self-completed questionnaires translated in English.

**Additional file 2:** **Supplementary Table 1. **Distribution of sample for each year, type of school and number of smokers in each type of smokers.

## Data Availability

The datasets used and/or analysed during the current study are available from corresponding author (murielle.mary-krause@iplesp.upmc.fr) on reasonable request, and with permission of the executive committee of Paris sans Tabac and Paris Rectorat.

## Abbreviations

e-cigarette: Electronic cigarette
OR: Odds-ratio
aOR: Adjusted odds-ratio
95% CI: 95% Confidence interval
PST: Paris sans tabac [Paris without tobacco]
AUC: Area under Roc curve

## References

[1] Grana R, Benowitz N, Glantz SA (2014). E-cigarettes: a scientific review. Circulation.

[2] Pepper JK, Brewer NT (2014). Electronic nicotine delivery system (electronic cigarette) awareness, use, reactions and beliefs: a systematic review. Tob Control.

[3] Miech R, Johnston L, O’Malley PM, Bachman JG, Patrick ME (2019). Trends in adolescent vaping, 2017–2019. N Engl J Med.

[4] Dutra LM, Glantz SA (2014). High international electronic cigarette use among never smoker adolescents. J Adolesc Health.

[5] Goniewicz ML, Gawron M, Nadolska J, Balwicki L, Sobczak A (2014). Rise in electronic cigarette use among adolescents in Poland. J Adolesc Health.

[6] ESPAD Report 2019: results from the European School Survey Project on Alcohol and Other Drugs, EMCDDA joint publications, publications office of the European Union, Luxembourg, 2020. http://espad.org/sites/espad.org/files/2020.3878_EN_04.pdf. Accessed on 26 Jan2022.

[7] Andler R, Guignard R, Wilquin JL, Beck F, Richard JB, Nguyen-Thanh V (2016). Electronic cigarette use in France in 2014. Int J Public Health.

[8] Pasquereau A, Gautier A, Andler R, Guignard R, Richard JB, Nguyen-Thanh V (2017). Tabac et e-cigarette en France : Niveaux d’usage d’après les premiers résultats du baromètre santé 2016 [Tobacco and e-cigarette in France: Usage levels according to the first results of the 2016 Health Barometer]. Bull Epidémiol Hebd.

[9] Chyderiotis S, Spilka S, Beck F (2019). Usages de la cigarette électronique en France à 17 ans : résultats de l’enquête nationale ESCAPAD 2017 [Use of electronic cigarette in France among adolescents aged 17: Results from the ESCAPAD 2017 survey]. Bull Cancer.

[10] Johnston LD, O'Malley PM, Miech RA, Bachman JG, Schulenberg JE (2016). Monitoring the Future national survey results on drug use: 1975–2015: Overview, key findings on adolescent drug use.

[11] Sun R, Mendez D, Warner KE. Can PATH study susceptibility measures predict e-cigarette and ciogarette use among American youth one year later. Addiction. 2022. 10.1111/JCN.0000000000add.15808. 10.1111/add.15808 35072302

[12] Lee YO, Kim AE (2015). ‘Vape shops’ and ‘E-Cigarette lounges’ open across the USA to promote ENDS. Tob Control.

[13] Sutfin EL, Reboussin BA, Debinski B, Wagoner KG, Spangler J, Wolfson M (2015). The impact of trying electronic cigarettes on cigarette smoking by college students: a prospective analysis. Am J Public Health.

[14] Escobedo LG, Reddy M, DuRant RH (1997). Relationship between cigarette smoking and health risk and problem behaviors among US adolescents. Arch Pediatr Adolesc Med.

[15] Jessor R (1991). Risk behavior in adolescence: A psychosocial framework for understanding and action. J Adolesc Health.

[16] Jessor R (1993). Successful adolescent development among youth in high-risk settings. Am Psychol.

[17] Camenga DR, Kong G, Cavallo DA (2014). Alternate tobacco product and drug use among adolescents who use electronic cigarettes, cigarettes only, and never smokers. J Adolesc Health.

[18] Hughes K, Bellis MA, Hardcastle KA (2015). Associations between e-cigarette access and smoking and drinking behaviours in teenagers. BMC Public Health.

[19] Kristjansson AL, Mann MJ, Sigfusdottir ID (2015). Licit and illicit substance use by adolescent e-cigarette users compared with conventional cigarette smokers, dual users, and nonusers. J Adolesc Health.

[20] Miech RA, O'Malley PM, Johnston LD, Patrick ME (2016). E-cigarettes and the drug use patterns of adolescents. Nicotine Tob Res.

[21] Soneji S, Barrington-Trimis JL, Wills TA (2017). Association between initial use of e-cigarettes and subsequent cigarette smoking in adolescents and young adults. A systematic review and meta-analysis. JAMA Pediatr.

[22] Conner M, Grogan S, Simms-Ellis R (2017). Do electronic cigarettes increase cigarette smoking in UK adolescents? Evidence from a 12-month prospective study. Tob Control.

[23] Best C, Haseen F, Currie D (2017). Relationship between trying an electronic cigarette and subsequent cigarette experimentation in Scottish adolescents: a cohort study. Tob Control.

[24] Chan GCK, Stjepanovic D, Lim C, Sun T, Shanmuga Anandan A, Connor JP, Gartner C, Hall WD, Leung J (2020). Gateway or common liability ? A systematic review and meta-analysis of studies of adolescent e-cigarette use and future smoking initiation. Addiction.

[25] Foxon F, Selya AS (2020). Electronic cigarettes, nicotine use trends and use initiation ages among US adolescents from 1999 to 2018. Addiction.

[26] Pasquereau A, Quatremère G, Guignard R, et al. Baromètre de Santé Publique France 2017. Usage de la cigarette électronique, tabagisme et opinions des 18–75 ans [2017 Health Barometer of France Public Health. Electronic cigarette use, smoking and opinions of 18–75 year-olds]. Ed Santé Publique France, Saint-Maurice, France, 2019. https://www.santepubliquefrance.fr/determinants-de-sante/tabac/documents/enquetes-etudes/barometre-de-sante-publique-france-2017.-usage-de-la-cigarette-electronique-tabagisme-et-opinions-des-18-75-ans. Accessed on 14 April 2022.

[27] Sompa SI, Zettergren A, Ekström S, Upadhyay S, Ganguly K, Georgelis A, Ljungman P, Pershagen G, Kull I, Melén E, Palmberg L, Bergström A (2022). Predictors of electronic cigarette use and its association with repiratory health and obesity in young adulthood in Sweden; findings from the population-based birth cohort BAMSE. Environ Res.

[28] Johnston LD, O'Malley PM, Bachman JG, Schulenberg JE (2012). Monitoring the future national survey results on drug use: 1975–2011: volume I, secondary school students.

[29] Gentzke AS, Wang TW, Cornelius M, Park-Lee E, Ren G, Sawdey MD, Cullen KA, Loretan C, Jamal A, Homa DM (2022). Tobacco product use and associated factors among middle and high school students – National Youth Tobacco Survey, United Stated, 2021. MMWR Surveill Summ.

[30] Spilka S, Le Nézet O, Beck F, Ehlinger V, Godeau E (2012). Alcool, tabac et cannabis durant les «années collège». Tendances..

[31] Le Nézet O, Ngantcha M, Beck F, Spilka S (2016). La consommation de tabac au cours des années lycée Résultats de l’enquête ESPAD 2015 [Tobacco use among French high-school students in 2015Results from the 2015 European scool survey project on alcohol and other druigs (ESPAD)]. Bull Epidémiol Hebd.

[32] Spilka S, Le Nézet O, Tovar ML (2012). Les drogues à 17 ans : premiers résultats de l’enquête ESCAPAD 2011. Tendances.

[33] Peretti-Wadel P, Legleye S, Guignard R, Beck F (2014). Cigarette smoking as a stigma: evidence from France. Int J Drug Policy.

[34] Rubin D (1987). Multiple imputation for nonresponse in surveys.

[35] Lohr SL (2009). Sampling: design and analysis.. Advanced series.

[36] Hedecker D (2003). A mixed-effects multinomial logistic regression model. Stat Med.

[37] Tibshirani R (1996). Regression shrinkage and selection via the lasso. J R Statist Soc B.

[38] Zhong J, Cao S, Gong W, Fei F, Wang M (2016). Electronic cigarettes use and intention to cigarette smoking among never-smoking adolescents and young adults: a meta-analysis. Int J Environ Res Public Health.

[39] Leventhal AM, Stone MD, Andrabi N (2016). Association of e-cigarette vaping and progression to heavier patterns of cigarette smoking. JAMA.

[40] King JL, Reboussin BA, Wiseman KD, Ribisl KM, Seidenberg AB, Wagoner KG, Wolfson M, Sutfin EL (2019). Adverse symptoms users attribute to e-cigarettes: Results from a national survey of US adults. Drug Alcohol Depend.

[41] Li D, Sundar IK, McIntosh S, Ossip DJ, Goniewicz ML, O’Connor RJ, Rahman I (2020). Association of smoking and electronic cigarette use with wheezing and related respiratory symptoms in adults: cross-sectional results from the Population Assessment of Tobacco and Health (PATH) study, wave 2. Tob Control.

[42] Osei AD, Mirbolouk M, Orimoloye OA, Dzaye O, Uddin SMI, Benjamin EJ, Hall ME, DeFilippis AP, Stokes A, Bhatnager A, Nasir K, Blaha MJ (2019). Association between e-cigarette use and cardiovascular disease among never and current combustible-cigarette smokers. Am J Med.

[43] Berthier N, Guignard R, Richard JB, Andler R, Beck R, Nguyen-Thanh V (2016). Comparaison régionale du tabagisme et de l’usage de cigarette électronique en France en 2014 [Regional comparison of smoking and e-cigarette use in France in 2014]. Bull Epidemiol Hebd.

[44] Institut national de la statistique et des études économiques (INSEE). Dossier complet Département de Paris (75) [Complete file Paris department]. https://www.insee.fr/fr/statistiques/2011101?geo=DEP-75. Accessed on 14 April 2022.

[45] Vaughn MG, Salas-Wright CP, Kremer KP, Maynard BR, Roberts G, Vaughn S (2015). Are homeschooled adolescents less likely to use alcohol, tobacco, and other drugs?. Drug Alcohol Depend.

[46] U.S. Department of Health and Human Services (2016). E-Cigarette use among youth and young adults. A report of the Surgeon General.

[47] O’Malley PM, Bachman JG, Johnston LD (1983). Reliability and consistency in self-reports of drug use. Int J Addict.

[48] Harrison L (1997). The validity of self-reported drug use in survey research: An overview and critique of research method. NIDA Res Monogr.

[49] Spindle TR, Hiler MM, Cooke ME, Eissenberg ME, Kendler KS, Dick DM (2017). Electronic cigarette use and uptake of cigarette smoking: a longitudinal examination of U.S. college students. Addict Behav.

[50] Kong G, Morean ME, Cavallo DA, Camenga DR, Krishnan-Sarin S (2015). Reasons for electronic cigarette experimentation and discontinuation among adolescents and young adults. Nicotine Tob Res.

[51] Pepper JK, Ribisl KM, Emery SL, Brewer NT (2014). Reasons for starting and stopping electronic cigarette use. Int J Environ Res Public Health.

[52] Tremblay B, Turk MT, Cooper MR, Zoucha R (2020). Knowledge, attitudes, and perceptions of young adults about electronic nicotine delivery systems in the United States: An integrative review. J Cardiovasc Nurs.

[53] Wills TA, Knight R, Williams RJ, Pagano I, Sargent JD (2015). Risk factors for exclusive e-cigarette use and dual e-cigarette and tobacco use in adolescents. Pediatrics.

[54] Leventhal AM, Strong DR, Sussman S (2016). Psychiatric comorbidity in adolescent electronic and conventional cigarette use. J Psychiat Res.

[55] Jeon C, Jung KJ, Kimm H (2016). E-cigarettes, conventional cigarettes, and dual use in Korean adolescents and university students: prevalence and risk factors. Drug Alcohol Depend.

[56] McCabe SE, Veliz P, McCabe VV, Boyd CJ (2017). Smoking behaviors and intentions among current e-cigarette users, cigarette smokers, and dual users: a national survey of U.S. high school seniors. Prev Med.

[57] McCabe SE, West BT, Veliz P, Boyd CJ (2017). E-cigarette use, cigarette smoking, dual use, and problem behaviors among U.S. adolescents: results from a National survey. J Adolesc Health.

[58] Kaufmann N, Currie D (2017). The Scottish adolescent e-cigarette user: profiling from the Scottish Schools Adolescent Lifestyle and Substance Use Survey (SALSUS). Public Health.

[59] Rainio SU, Rimpelä AJ (2008). Home smoking bans in Finland and the association with child smoking. Eur J Public Health.

[60] Emory K, Saquib N, Gilpin EA, Pierce JP (2010). The association between home smoking restrictions and youth smoking behavior: a review. Tob Control.

[61] Loan CM, Khurana A, Wright J, Romer D (2021). Selection versus socialization effects of peer norms on adolescent cigarette use. Tob Use Insights.

[62] Deutsch AR, Chernyavskiy P, Steinley D, Slutske WS (2105). Measuring peer socialization for adolescent substance use: a comparison of perceived and actual friends’ substance use effects. J Stud Alcohol Drugs.

[63] Maziak W, Taleb ZB, Bahelah R, Islam F, Jaber R, Auf R, Salloum RG (2015). The global epidemiology of water-pipe smoking. Tob Control.

[64] Wang TW, Gentzke A, Sharapova S, Cullent KA, Bridget KA, Jamal A (2018). Tobacco product use among middle and high school students – United States, 2011–2017. MMWR Morb Mortal Wkly Rep.

[65] Andler R, Guignard R, Wilquin JL, Beck F, Nguyen-Thanh V (2015). L’usage de la cigarette électronique en France en 2014 [Electronic cigarette use in France in 2014]. Evolutions.

[66] Tann J, Warner KE (2018). Students’ cigarette smoking and the perceived nicotine contents of their e-cigarettes. Am J Prev Med.

[67] Wang TW, Gentzke AS, Creamer MR, Cullen KA, Holder-Hayes E, Sawdey MD, Anic GM, Portnoy DB, Hu S, Jamal A, Neff LJ (2019). Tobacco product Use and associated factors among middle and high school students- United States, 2019. MMWR Surveill Summ.

[68] Observatoire Français des Drogues et des Toxicomanies (2019). Drogues, chiffres clés.

[69] Spilka S, Godeau E, Le Nézet O, Ehlinger V, Janssen E, Brissot A, Philippon A, Chyderiotis S (2019). Usages d’alcool, de tabac et de cannabis chez les adolescents du secondaire en 2018. Tendances.

[70] Overbeek DL, Kass AP, Chiel LE, Boyer EW, Casey AMH (2020). A review of toxic effets of electronic cigarettes/vaping in adolescents and young adults. Crit Rev Toxicol.

