# Supplementary material for: What differentiates youths who use e-cigarettes from those who smoke traditional tobacco products?
Source: BMC Public Health. 2022 Jul 15;22:1357. doi: 10.1186/s12889-022-13673-0 (PMC9288082; doi:10.1186/s12889-022-13673-0)
Supplement: Supplementary file 1 — Additional file 1: Supplementary Figure 1. Students self-completed questionnaires translated in English. [file 12889_2022_13673_MOESM1_ESM.docx]

**Supplementary Figure 1**: Students self-completed questionnaires translated in English

# 2017 Survey coordinated by «Paris without Tobacco»

**Confidential and Anonymous questionnaire**

Birth year 🞎<1997 🞎97 🞎98 🞎99 🞎2000 🞎01 🞎02 🞎03 🞎04 🞎05 🞎06 🞎>2006

**Sex**

🞎 male

🞎 female

**Smokers around you a smoker** a **former-smoker a non-smoker**

Is your father? 🞎 🞎 🞎

Is your mother? 🞎 🞎 🞎

How many of your 4 best friends smoke? 🞎0 🞎1 🞎2 🞎 3 🞎4

Do you have any sisters or brothers who smoke?

🞎Yes 🞎No 🞎I am an only child

**What do you think about tobacco ?**

It is a pleasure 🞎Yes 🞎No

It is a trap (difficult to stop) 🞎Yes 🞎No

We smoke mostly by addiction 🞎Yes 🞎No

**Did you receive information about smoking in elementary school?** 🞎Yes 🞎No

**Did you receive information about smoking in middle school?** 🞎Yes 🞎No

**Have you heard about the campaign«*Me/month without tobacco»*?**

🞎No 🞎Yes, but I did not participate 🞎Yes, and I participated

**Do you ever drink alcohol?** 🞎Yes 🞎No

**All**

If yes 🞎<1 time/month 🞎2-4 times/month 🞎1 time/week 🞎2-3 times/week 🞎everyday

Have you ever had >4 drinks on the same day? 🞎 Never 🞎1-4 times 🞎>4 times

**Do your parents ask you not to smoke?** (only one answer)

🞎yes both 🞎yes my mother 🞎yes my father 🞎No

In your opinion, **in your class**, out of 10 students how many smoke every day?

🞎0 🞎1 🞎2 🞎3 🞎4 🞎5 🞎6 🞎7 🞎8 🞎9 🞎10

**How is your home?**

🞎strictly non-smoking 🞎It may happen that someone smokes inside 🞎you can smoke freely inside

**Have you ever consumed, even only once, even only one drag:**

a cigarette? 🞎yes 🞎no a water pipe (hookah)? 🞎yes 🞎no an electronic cigarette? 🞎yes 🞎no

Among these products, indicate which one you used first? PLEASE indicate **THE very first time only**

🞎 classic cigarette 🞎 menthol cigarette 🞎 do it yourself cigarette 🞎 water pipe

Single answer line

🞎 cigarillo 🞎 e-cigarette with nicotine 🞎 e-cigarette without nicotine

Are you currently? 🞎 non-smoker

🞎 former smoker (stop > 6 months)

 🞎 regular smoker (at least one cigarette or water pime/day)

🞎 occasional smoker (does not smoke daily)

In the preceding 30 days, have you used? (multiple replies are possible):

🞎 classic cigarette 🞎 menthol cigarette 🞎 flavoured cigarette 🞎 water pipe

🞎 water pipe 🞎 cigarillo 🞎 e-cigarette with nicotine

Have you ever smoked anything else than tobacco? 🞎 yes 🞎 no (if no, skip the «Cannabis» paragraph)

**If you answered yes to the above question, answer these 4 questions**

Age of cannabis onset? 🞎<10 years 🞎10 🞎11 🞎12 🞎13 🞎14 🞎15 🞎16 🞎17 🞎>17years

When was your last cannabis consumption?

**Cannabis**

🞎< 1/month 🞎1-6 /month 🞎2-6/week 🞎1-2/day 🞎3-5/day 🞎>5/day

Do you use a bang? 🞎 never 🞎 sometimes

Do you use: 🞎 resin (shit) 🞎 leaves (bud) 🞎 oil 🞎 synthetic cannabinoids

Are you considering stopping cannabis use ?

🞎 no 🞎 yes one day 🞎 yes within 6 months 🞎 yes in the month 🞎 I have already stopped

**If you are a tobacco smoker (occasional or daily), answer these questions**

**Smokers**

On average, how many cigarettes do you currently smoke per day?

 🞎<1 🞎1 🞎2 🞎3 🞎4 🞎5 🞎6 🞎7 🞎8 🞎9 🞎10 🞎11-15 🞎16-20 🞎21-25 🞎>25/day

In the morning, how long after you wake up do you smoke your first cigarette ?

🞎 within 5 minutes 🞎 6-30 minutes 🞎 31-60 minutes 🞎 More than 60 minutes

A what age did you start smoking?

🞎<6 years 🞎6 🞎7 🞎8 🞎9 🞎10 🞎11 🞎12 🞎13 🞎14 🞎15 🞎16 🞎17 🞎18 🞎>18 years

Have you ever completely quit smoking? 🞎 no 🞎 1 times 🞎 2 times 🞎 >2 times

Are you considering quitting smoking?

🞎 no 🞎 yes one day 🞎 yes within 6 months 🞎 yes in the month 🞎 don’t know

In 2017, did you buy tobacco from a tobacco shop? 🞎 Yes 🞎 No

If you attempted to purchase tobacco, were you asked? :

Your age? 🞎 Yes 🞎 No

For your ID card ? 🞎 Yes 🞎 No

If you purchase tobacco, age of the 1^st^ purchase in a tobacco shop?

🞎<10 years 🞎11 🞎12 🞎13 🞎14 🞎15 🞎16 🞎17 🞎18 🞎>18 years

**If you ever tried an e-cigarette, please answer the following questions**

**Did you start with** 🞎 an e-cigarette? 🞎 tobacco?

**What was the first thing you stopped?** 🞎 tobacco 🞎 e-cigarette 🞎 I still use both

**Do you use an e-cigarette**

🞎 every day? 🞎 not every day, but >once per week? 🞎 1-4 times a month? 🞎 not this month?

**What flavouring do you use in e-cigarettes ?**

🞎 tobacco 🞎apple 🞎mint 🞎caramel 🞎liquorice 🞎vanilla

**What type of e-cigarette do you use?**

🞎e-cigarette for single use 🞎ready-to-use cartridges 🞎Mod Box

🞎pen-shaped 🞎e-cigarette with big battery

**A 10ml filler bottle lasts:**

**Vapers**

🞎1 day 🞎2 🞎3 🞎4 🞎5 🞎6 🞎7 🞎8 🞎9 🞎10 🞎11-30 days 🞎> 30 days 🞎I don’t use it

**Nicotine concentration currently used:**

🞎none 🞎2-4 mg/mL 🞎5-7 🞎8 🞎9 🞎10 🞎11 🞎12 🞎13 🞎14 🞎15-17 🞎18-20 mg/mL

**Do you add products to e-liquids?**

🞎no 🞎yes, essential oils 🞎yes, alcohol 🞎yes, cannabis 🞎yes, other, specify …………………………………

**Where do you buy e-liquids and products?**

🞎I don’t buy myself 🞎tobacco shop 🞎specialty store 🞎Internet 🞎other, specify …………………………..
