# Supplementary material for: What differentiates youths who use e-cigarettes from those who smoke traditional tobacco products?
Source: BMC Public Health. 2022 Jul 15;22:1357. doi: 10.1186/s12889-022-13673-0 (PMC9288082; doi:10.1186/s12889-022-13673-0)
Supplement: Supplementary file 2 — Additional file 2: Supplementary Table 1. Distribution of sample for each year, type of school and number of smokers in each type of smokers. [file 12889_2022_13673_MOESM2_ESM.docx]

**Supplementary Table 1**: Distribution of sample for each year, type of school and number of smokers in each type of smokers

|  |  | Non users | Exclusive e-cigarette users | Exclusive tobacco users | Dual users | TOTAL |
| --- | --- | --- | --- | --- | --- | --- |
| 2013 | Middle school | 1612  (93.5%) | 30  (1.7%) | 67  (3.9%) | 15  (0.9%) | 1724* |
|  | High school | 1178  (75.8%) | 13  (0.8%) | 280  (18.0%) | 84  (5.4%) | 1555* |
| 2014 | Middle school | 1171  (93.7%) | 51  (4.1%) | 13  (1.0%) | 15  (1.2%) | 1250* |
|  | High school | 1717  (81.3%) | 58  (2.7%) | 211  (10.0%) | 127  (6.0%) | 2113* |
| 2015 | Middle school | 1762  (93.9%) | 63  (3.3%) | 28  (1.5%) | 24  (1.3%) | 1877* |
|  | High school | 1266  (77.2%) | 33  (2.0%) | 245  (14.9%) | 96  (5.9%) | 1640* |
| 2016 | Middle school | 1733  (95.2%) | 38  (2.1%) | 32  (1.8%) | 17  (0.9%) | 1820* |
|  | High school | 1348  (78.0%) | 41  (2.4%) | 252  (14.6%) | 86  (5.0%) | 1727* |
| 2017 | Middle school | 1761  (96.3%) | 35  (1.9%) | 18  (1.0%) | 14  (0.8%) | 1828* |
|  | High school | 1570  (82.6%) | 30  (1.6%) | 224  (11.8%) | 77  (4.0%) | 1901* |
| TOTAL |  | 15118 | 392 | 1370 | 555 | 17435 |

* p value of the Chisquare <0.0001
